# Genetic ablation of fibroblast activation protein alpha attenuates left ventricular dilation after myocardial infarction

**DOI:** 10.1371/journal.pone.0248196

**Published:** 2021-03-05

**Authors:** Daniel B. Hoffmann, Daniela Fraccarollo, Paolo Galuppo, Stefan Frantz, Johann Bauersachs, Jochen Tillmanns

**Affiliations:** 1 Department of Trauma-, Orthopaedic- and Plastic Surgery, University Medical Center Göttingen, Göttingen, Germany; 2 Department of Cardiology and Angiology, Hannover Medical School, Hannover, Germany; 3 Department of Medicine, University Hospital Wurzburg, Wuerzburg, Germany; Max Delbruck Centrum fur Molekulare Medizin Berlin Buch, GERMANY

## Abstract

**Introduction:**

Regulating excessive activation of fibroblasts may be a promising target to optimize extracellular matrix deposition and myocardial stiffness. Fibroblast activation protein alpha (FAP) is upregulated in activated fibroblasts after myocardial infarction (MI), and alters fibroblast migration *in vitro*. We hypothesized that FAP depletion may have a protective effect on left ventricular (LV) remodeling after MI.

**Materials and methods:**

We used the model of chronic MI in homozygous FAP deficient mice (FAP-KO, n = 51) and wild type mice (WT, n = 55) to analyze wound healing by monocyte and myofibroblast infiltration. Heart function and remodeling was studied by echocardiography, morphometric analyses including capillary density and myocyte size, collagen content and in vivo cell-proliferation. In non-operated healthy mice up to 6 months of age, morphometric analyses and collagen content was assessed (WT n = 10, FAP-KO n = 19).

**Results:**

Healthy FAP-deficient mice did not show changes in LV structure or differences in collagen content or cardiac morphology. Infarct size, survival and cardiac function were not different between FAP-KO and wildtype mice. FAP-KO animals showed less LV-dilation and a thicker scar, accompanied by a trend towards lower collagen content. Wound healing, assessed by infiltration with inflammatory cells and myofibroblasts were not different between groups.

**Conclusion:**

We show that genetic ablation of FAP does not impair cardiac wound healing, and attenuates LV dilation after MI in mice. FAP seems dispensable for normal cardiac function and homeostasis.

## Introduction

The myocardial extracellular matrix (ECM) is a critical component in normal and pathophysiological conditions of the heart, and is mainly regulated by cardiac fibroblasts [1]. During early ventricular remodeling after myocardial infarction (MI) the invasion and activity of myofibroblasts is critical for wound healing and scar development [2, 3]. However, elevated deposition of ECM by fibroblasts leads to cardiac dysfunction in late remodeling [4]. Protecting the left ventricle (LV) from detrimental remodeling after MI is still a challenge. Even if the current medical treatment options after MI including RAAS inhibitors, mineralocorticoid antagonists and beta blockers show beneficial effects, there is still no specific antifibrotic treatment option for chronic ventricular remodeling [4].

The dipeptidyl-peptidase *Fibroblast activation protein α* (FAP) is a serine protease expressed by activated fibroblasts after MI in animals and humans [5, 6]. FAP is highly expressed in activated fibroblasts by a TGFβ-driven mechanism after MI in rats, and promotes fibroblast migration and exerts gelatinolytic activity *in vitro* [5]. FAP ^pos^ activated fibroblasts are present in hearts of patients with chronic ischemic cardiomyopathy, demonstrating persistent fibrotic activity in these patients [5]. FAP was also detected in human atherosclerotic plaques and associated with plaque progression and fibrous cap thinning [7], whereas deletion of FAP decreased atherosclerotic plaque formation in a mouse model [8]. Increased expression of FAP was also found in pathological fibrotic diseases like idiopathic pulmonary fibrosis [9], liver cirrhosis [10] and keloids [11] as well as in stromal soft tissue of several kinds of cancer [12–14]. In healthy hearts and other tissues the expression of FAP is absent or very low [12, 15].

A first successful attempt has been described to reduce cardiac fibrosis by targeting FAP-expressing fibroblasts in rodents using antigen-specific CD8^pos^ T cells in angiotensin II/phenylephrine induced myocardial fibrosis [16]. Because therapies targeting FAP^pos^ myofibroblasts will also alter myocardial FAP levels, it is important to understand the function and pathophysiological significance of FAP deficiency in normal healthy hearts and post-MI *in vivo*. Since FAP is upregulated in fibrotic diseases and especially after MI and alters fibroblast migration, we hypothesized that FAP depletion may have a protective effect on LV remodeling after MI.

## Materials and methods

Additional materials and methods are presented as Supplementary Online Material.

### Experimental myocardial infarction in mice

Myocardial infarction (MI) was induced in female homozygous FAP-deficient mice (FAP-KO, n = 51) on a C57BL/6NCrl background [17], generated by Niedermeyer et al. [18], or wildtype C57BL/6NCrl mice (n = 55) aged 12 weeks as described previously [19, 20]. Briefly, under 1.5–2% isoflurane anesthesia (induction with 5% isoflurane), the thorax was opened and the proximal left anterior descending coronary artery was occluded using a 5–0 suture. Animals were kept warm with a heating pad. Depth of anesthesia was tested using the pedal withdrawal reflex. Analgesia was maintained using buprenorphine (0.05 mg/kg BW i.p.). Before and after surgery, animals were housed in the animal facility and monitored daily for activity and signs of pain. Surviving animals were euthanized by cardiac arrest using intracardiac injection of saturated potassium chloride solution and hearts removed at 7 days and 4 weeks after MI. An additional group of mice was studied without surgery for six months to assess physiological changes in animals (WT n = 10, FAP-KO n = 19). The hearts were removed for anatomical, histological and western blot analyses and fixed with paraformaldehyde or frozen. Short term survival analysis was performed in a subgroup of 56 operated animals (WT operated n = 26, surviving n = 12; FAP-KO n = 30, surviving n = 12). Analysis for ventricular rupture was performed by macroscopic inspection in a subgroup of WT (n = 11) and FAP KO (n = 13) operated animals. Animal studies were conducted in accordance with the principles and procedures outlined in the Guide for the Care and Use of Laboratory Animals and were approved by the local government (Regierung von Unterfranken permission number K 55.2–2531.01-64/09).

### Echocardiographic analysis

We performed serial transthoracic echocardiography at days 1, 14 and 28 after MI by an experienced technician as described previously by Vogel et al. [21]. Echocardiography was performed under isoflurane anesthesia and spontaneous respiration. The endocardial borders were traced at end-systole and end-diastole with the help of a prototype analysis off-line system (NICE; Toshiba Medical System, Netherlands). Parameters were measured at the mid-papillary and apical muscle level in B-Mode images.

### Immunohistochemistry of mouse myocardial tissue

Frozen or formalin-fixed paraffin-embedded sections from mouse hearts were stained with antibodies against CD68, α smooth muscle actin (SMA) and CD31 and quantified as described in S1 Material. At 28 days after MI, hearts were analyzed for the number of CD31^pos^ capillaries. For analysis of myocyte size, H&E stained formalin fixed paraffin embedded sections were imaged at 20x magnification, and areas of cross sectioned myocytes were analyzed in the intact myocardium using Image Pro Plus software (Media Cybernetics, Bethesda, USA).

### Analysis of cell proliferation in vivo

For detection of myocardial cell proliferation in vivo, MI was induced in female WT and FAP-KO mice at 12 weeks of age (n = 4 each). BrdU (Roche), 50 mg per kg body weight, was injected twice a day every day before sacrifice on day 14. For immunofluorescent staining of BrdU, formalin fixed, paraffin embedded tissues were sectioned at 4 μm, and heat induced antigen retrieval was performed using Histosafe Enhancer (Linaris, Germany). After a blocking-step using 10% donkey serum in PBS, sections were sequentially incubated with primary antibodies against BrdU (5-Bromo-2deoxy-uridine Labeling and detection kit, Roche) and detected by fluorescent secondary antibodies (Jackson ImmunoResearch). Nuclear DNA was labeled using DAPI (Invitrogen). Images were obtained at 20x with a Nikon NiE microscope, quantitative analysis of BrdU-positive and BrdU-negative nuclei was performed in the scar and the surviving free wall adjacent to the scar using Image Pro Plus software (Media Cybernetics, Bethesda, USA), and proliferation index was calculated. Image processing with Photoshop (Adobe) included changes in brightness, contrast and tonal range, and was applied equally across the entire image.

### Analysis of collagen content after MI and in healthy mice

Collagen content in the intact myocardium was analyzed 28d after MI by analyzing picrosirius red stained tissues sections. In healthy mice 6 months of age, collagen content was measured by use of hydroxyproline assay.

### Statistics

Data are presented as mean ± SE. We used the Mann-Whitney-U test for analysis of differences between two groups. Differences in ventricular ruptures between groups were analyzed by Fisher’s exact test. A value of P less than 0.05 was considered statistically significant. Statistical analysis was performed with Prism 5 (GraphPad).

## Results

### FAP deficiency does not impact LV morphology and fibrosis in healthy mice

We performed histological analyses in nonoperated healthy mice up to the age of six months. No differences between FAP-KO mice and WT mice in body weight, heart weight, LV morphology were observed (S1 Fig). Of note, collagen content, as measured by hydroxyproline assay, was similar in both groups indicating unaltered collagen homeostasis in healthy mice up to 6 months of age. Moreover, isolated fibroblasts from hearts of WT and FAP-KO mice showed no overt difference in phenotype and growth properties (S2 Fig).

### LV dilation is reduced in FAP deficient animals after MI

**Fig 1:**
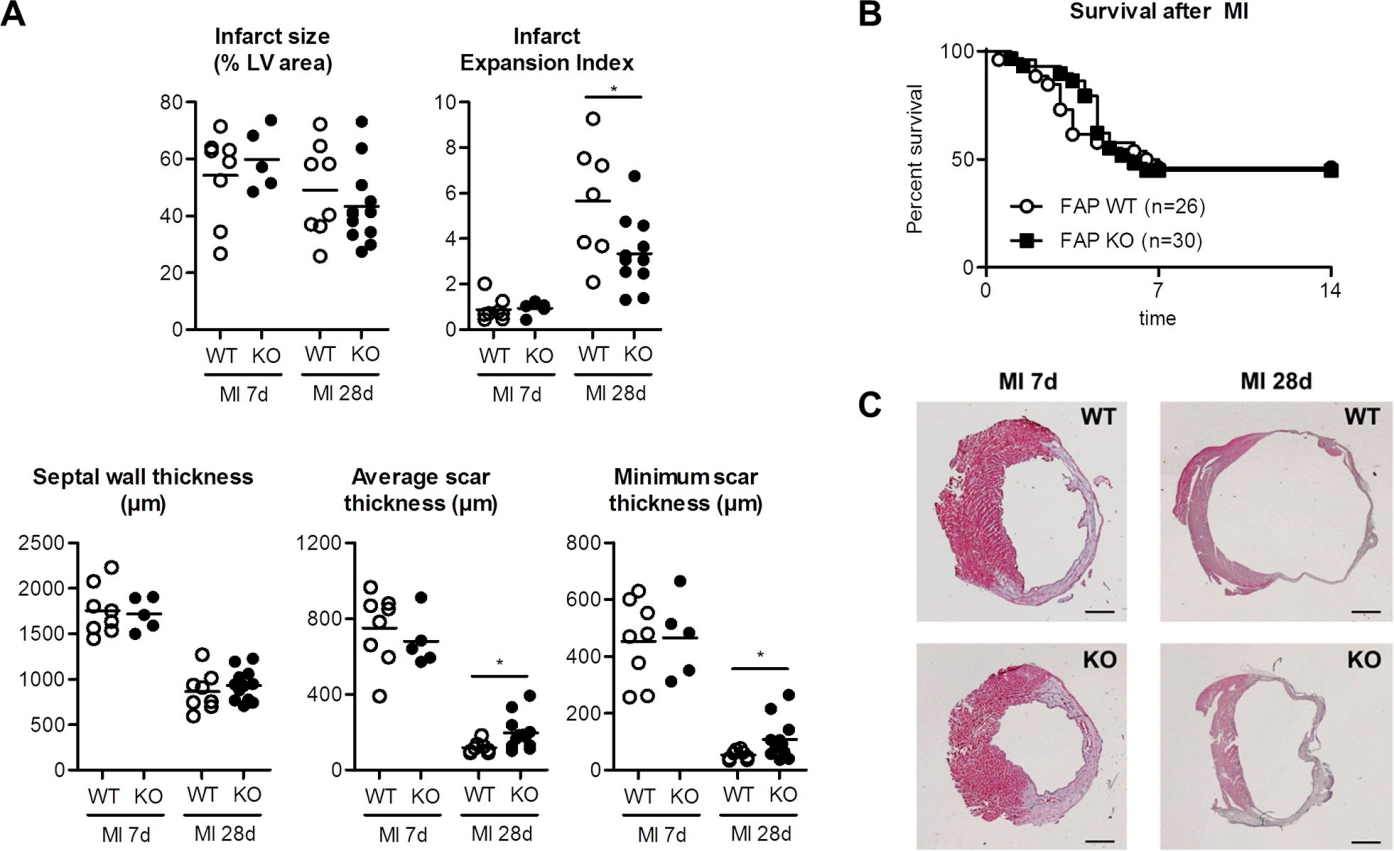
Infarct size, anatomical and morphometric measurements in FAP-KO and WT mice 7 days (WT: n = 8, FAP-KO: n = 5) and 28 days (WT: n = 8, FAP-KO: n = 12) after MI. A) Myocardial infarction (MI) size was not different between groups after 7 or 28 days. At 28 days after MI, average scar thickness and minimal scar thickness was higher in FAP-KO animals, resulting in reduced infarct expansion index compared to WT. B) Survival after MI was not different in both groups in the first 14 days. C) H&E stained sections of the LV 7d (left panels) and 28d (right panels) after MI. * = p<0.05 WT vs. FAP-KO. Plots show individual data and mean. Mann-Whitney-U test. Scale bars = 1mm.

We used the established model of chronic occlusion of the left coronary artery to induce large MI. Infarct size did not differ significantly between the FAP-KO group and WT group 7 and 28 days after MI (Fig 1A). Postoperative survival rate was similar in FAP-KO and WT mice two weeks after MI (WT: 50%, FAP-KO: 45%, n.s., Fig 1B). No difference in number of ventricular ruptures between FAP-KO and WT mice was observed in a subgroup of infarcted mice: Ventricular rupture was assumed in 5 out of 11 (45%) infarcted WT animals, and in 5 out of 13 (39%) infarcted FAP-KO animals (n.s.). FAP deficiency in FAP-KO animals was confirmed by western blot in infarcted hearts and in isolated cardiac fibroblasts from normal healthy hearts (S2A and S2B Fig).

To evaluate anatomical changes after MI, we performed morphometric analyses in transversal sections of infarcted hearts. At 7 days after MI, morphometric parameters were not different between groups (Fig 1A). In contrast, at 28 days after MI minimum and average scar thickness were about 70% larger in the FAP-KO group compared to the WT group (p<0.05). Accordingly, infarct expansion index was decreased in FAP-KO animals (WT: 5.7±0.6, FAP-KO: 3.5±0.3, p<0.05). No differences were observed in LV area (WT: 8.2±0.6mm^2^, FAP-KO: 8.8±0.6mm^2^, n.s.), LV cavity area (WT: 18.4±2.3mm^2^, FAP-KO: 14.6±1.2mm^2^, n.s.) and septum thickness after MI at 28 days after MI (Fig 1A). Body weight, wet lung weight, LV weight and RV weights did not show any difference between groups (S3 Fig).

**Fig 2:**
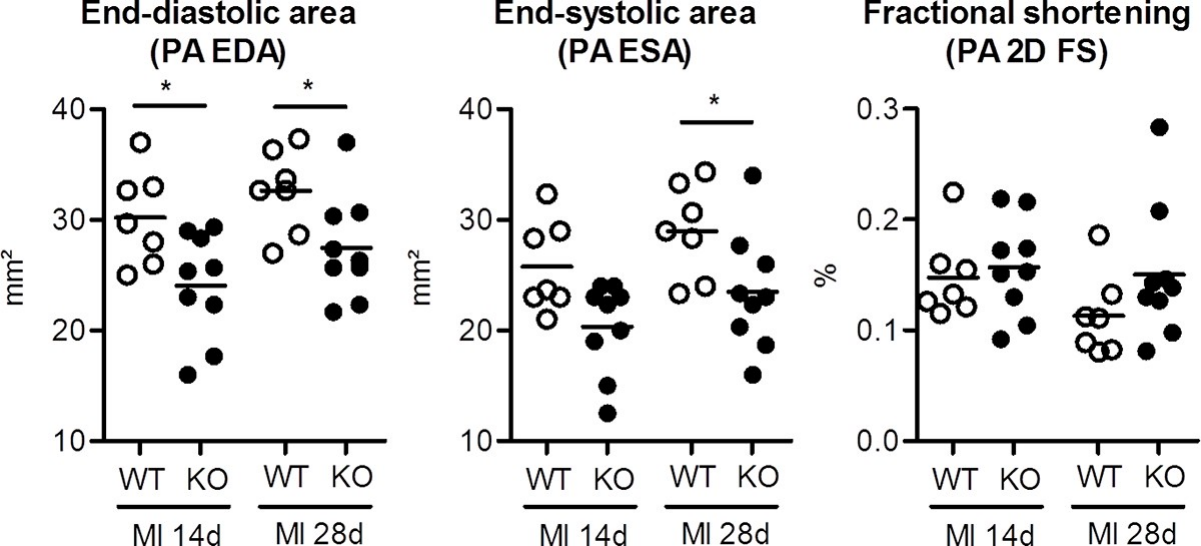
Echocardiography in FAP-KO and WT mice 14 and 28 days after MI. Left ventricular end-diastolic (PA EDA) and end-systolic area (PA ESA) measured at the papillary level was reduced in FAP-KO animals as compared to wild-type animals. Moreover, there was a trend towards increased fractional shortening (PA 2D FS) at papillary level in the FAP-KO group. * = p<0.05 WT vs. FAP-KO. Mann-Whitney-U test. Plots show individual data and mean. WT: n = 8, FAP-KO: n = 12.

We performed echocardiographic analyses to examine functional effects of FAP deficiency at days 1, 14 and 28 after MI (Fig 2; S1 Table). Corresponding to the reduced infarct expansion index in FAP-KO animals, end-diastolic area at papillary muscle level was decreased at 14 days (-21%, p<0.05) and 28 days (-17%, p<0.05) after MI in FAP-KO animals as compared to WT animals. Furthermore, end-systolic area at the papillary muscle level was also decreased 28 days after MI in FAP-KO animals (-21%, p<0.05). In agreement with the previous findings a trend towards reduced end-systolic and end-diastolic LV area was also detected when measured at the LV apical levels (S1 Table). LV systolic function as measured by fractional shortening was not different between groups at both time points.

Together, these results demonstrate improved LV remodeling by reduced LV dilation in FAP-KO animals at 28 days after MI.

### Monocyte and fibroblast infiltration are not altered in FAP deficient mice after MI

**Fig 3:**
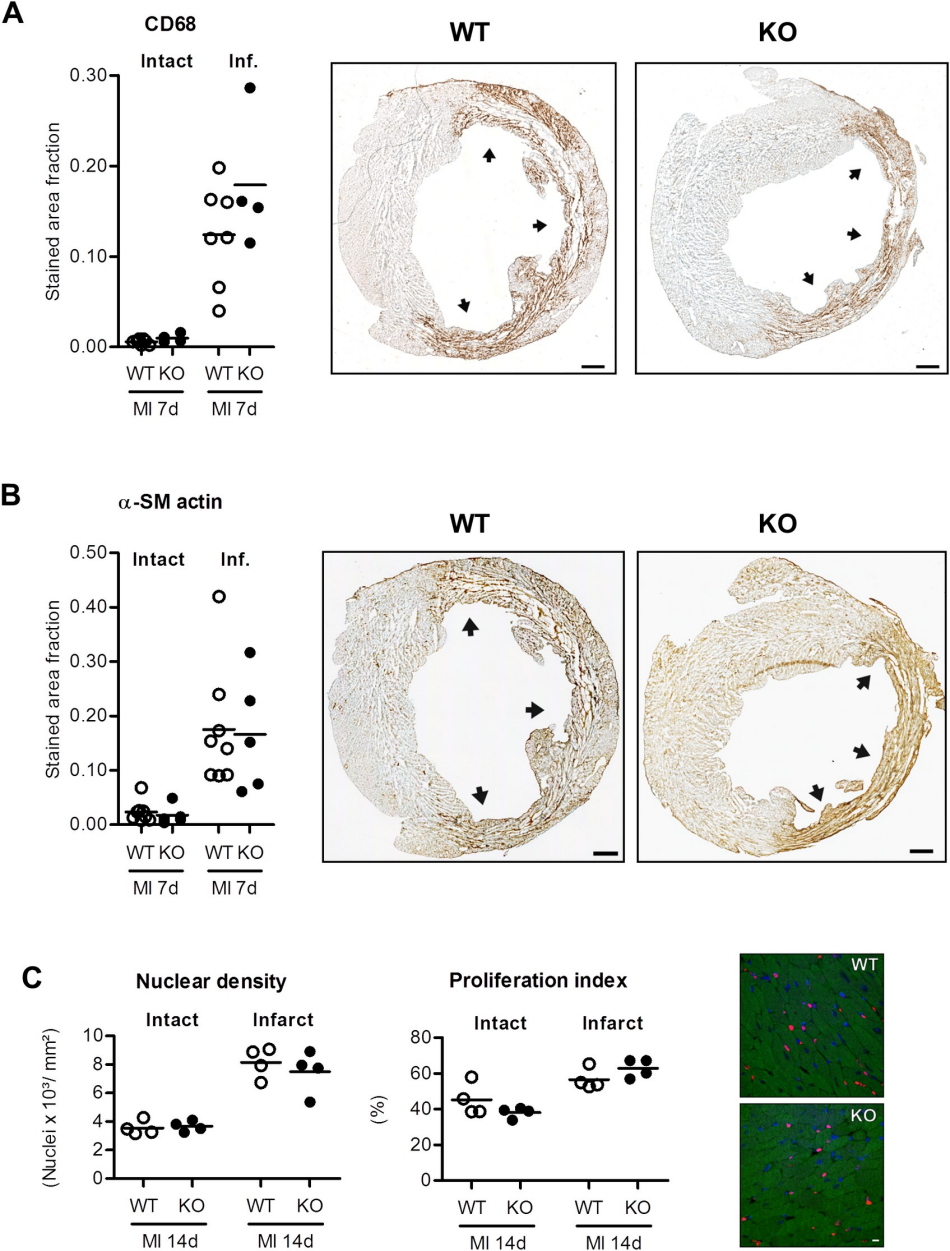
Quantitative analysis of stained area fractions of CD68 (A) and α-SM actin (B) in the intact and infarcted myocardium (inf.) at 7 days after myocardial infarction (MI). In the intact myocardium, CD68 and α-SM actin expression was very low. In contrast, infarcted myocardium showed robust expression of both CD68 and alpha-SM actin, with no significant difference between both groups. Representative LV sections shown in A (CD68) and B (α-SM actin) are from the same animal in WT (left panels) or FAP-KO group (right panels), respectively. C) Nuclear density and proliferation index were analyzed in animals treated with twice daily BrdU injections for 14 days after MI. While there was increased nuclear density and proliferation in the infarcted myocardium compared to intact myocardium at 14 days after MI, no differences between nuclear density or proliferation index in WT vs. FAP-KO animals were detected (n.s.). Plots show individual data and mean. A: WT n = 7, FAP-KO n = 4; B: WT n = 8, FAP-KO n = 5; C: WT n = 3, FAP-KO n = 4; Mann-Whitney-U test. Scale bars = 500μm (A,B) or 10μm (C).

To understand possible mechanisms responsible for improved LV remodeling in FAP-KO mice, we analyzed the myocardium by immunohistochemistry at 7 days after MI. In both groups, infarcted myocardium was infiltrated with SMA^pos^ myofibroblasts and CD68^pos^ monocytes at 7d after MI, and no difference in expression of both markers was apparent between groups (Fig 3A and 3B). Additionally, total cell density and cell proliferation in intact or infarcted myocardium was not different between groups at 14d after MI, respectively (Fig 3C), indicating integrity of the cellular wound healing response after MI in FAP-KO mice.

**Fig 4:**
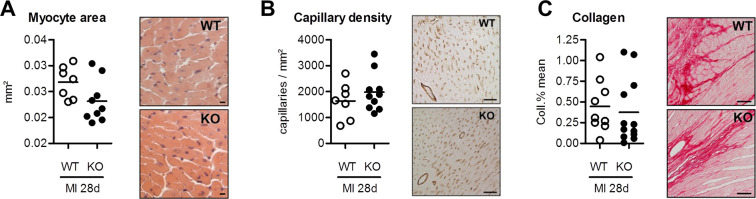
Quantitative analysis of myocyte cross sectional area (A), capillary density (B) and collagen content (C) in surving myocardium at 28 days after myocardial infarction (MI), showing no difference between groups. Plots show individual data and mean. A: WT n = 7, FAP-KO n = 9; B: WT n = 7, FAP-KO n = 10; C: WT n = 8, FAP-KO n = 12; Mann-Whitney-U test. Scale bars = 10μm (A), 50μm (B), 20μm (C).

Next, we analyzed the intact myocardium by immunohistochemistry at 28 days after MI to evaluate possible effects of FAP deficiency on myocytes, capillaries and collagen content. There were no differences in myocyte cross sectional area as well as capillary density in FAP-KO and WT mice (Fig 4). Collagen content, as measured by picrosirius red staining, showed a modest trend towards less collagen deposition in the FAP-KO group (n.s.).

Together, the morphometric results indicate unaltered inflammatory and fibroblast infiltration at the early wound healing phase, and a slight trend towards less collagen deposition in the chronic remodeling phase after MI in FAP-KO animals.

## Discussion

The serine protease FAP is upregulated after MI and primarily identifies activated myocardial fibroblasts [5, 6], but so far no studies have analyzed the physiological role of FAP in the heart *in vivo*. In this study, we show that genetic ablation of FAP does not alter cardiac wound healing but attenuates LV dilation after MI in mice.

### FAP deficiency attenuates LV dilation without affecting collagen content after MI

We analyzed the role of FAP on LV geometry and scar morphology after MI and show that the minimal and average scar thickness was greater in FAP deficient animals as compared to wildtype animals, thus attenuating LV dilation after 28 days. This data was also supported by echocardiography showing less LV dilation in FAP deficient animals. At the same time, animals did not show differences in signs of heart failure as body weight, heart and lung weights were not different in both groups.

Adverse cardiac remodeling with LV enlargement determines clinical impairment and mortality [22]. Therefore, improvement of cardiac remodeling is one of the main aims of current heart failure therapy [4]. LV morphology and dilation is dependent on collagen accumulation and structure, and balanced matrix degradation and production is a hallmark of post-MI wound healing. Matrix degradation is primarily performed by enzymes such as matrix metalloproteinases (MMP). In fact, inhibition of MMP activity has been studied extensively and shown to improve myocardial remodeling [23]. Genetic deletion of MMP-9 improved LV remodeling after MI [24]. Instead, cardiac overexpression of membrane type-1 matrix metalloproteinase (MT1-MMP) resulted in reduced LV function and increased fibrosis after MI [25]. In the study by Ducharme et al., LV dilation was accompanied by reduced collagen content and reduced inflammatory cell infiltration in MMP9-deficient mice after MI [24]. In comparison to these studies, we found only mild improvement in LV geometry in FAP-KO animals without difference in LV function, which might be a result of the only slightly reduced collagen content in the FAP-KO animals after MI, indicating that FAP has only minor effects on collagen homeostasis in the heart *in vivo* within the first 4 weeks after MI.

FAP is induced by TGFβ and TGFβ is one of the main profibrotic cytokines in the heart, highly upregulated after MI and necessary for improved wound healing and remodeling [5, 26, 27]. However, the reason why we did not find significant differences in collagen deposition between groups remains unclear. In this regard, a study using a TGFβ-overexpression model of chronic pulmonary fibrosis in mice also demonstrated only little effects of FAP deficiency on pulmonary fibrotic response [28]. This suggests in line with our results that TGFβ-induced tissue fibrosis is not mediated nor altered by FAP. Moreover, we demonstrated that 6 months old animals did not show any difference in LV morphology, indicating that FAP is not essential in normal myocardial homeostasis. This is further supported by data from Niedermayer et al. describing no developmental defects and normal heart morphology in FAP-deficient mice [18].

### Wound healing is not altered in FAP deficient animals

The initial wound healing phase after MI is critical for myocardial healing and paves the way for infarct repair [29], and depletion of monocytes/macrophages after MI leads to severely compromised extracellular matrix remodeling and increased infarct expansion [30]. In our study we found inflammatory cell infiltration, myofibroblast differentiation, overall cell density and cell proliferation to be not different between groups, indicating a normal wound healing response after MI [29]. These results suggest that FAP is not crucial for cell proliferation, adherence and migration within the myocardium after MI.

Of note, FAP is expressed in atherosclerotic plaques but its role in regulating inflammatory and fibrotic response is still poorly understood [7, 8, 31]. Two recent studies reported contrasting data: Monslow et al. demonstrated that global deletion of FAP in ApoE^−/−^ mice accelerated atherosclerotic disease progression by altering macrophage infiltration into the vulnerable plaque [31]. Instead, Stein et al. reported that deletion of FAP in ApoE^−/−^ mice resulted in decreased atherosclerotic plaque formation [8]. These diverging results show that while FAP has a role in atherosclerotic plaque progression, the mechanisms involved are not yet fully understood. Together with our findings we assume with reason that other factors, including well-known matrix degrading enzymes such as matrix-metalloproteinases are involved together with FAP in regulating myocardial ECM content and cell migration in the heart after MI, compensating the loss of FAP in the heart in FAP deficient mice [32].

### Study limitations

A main limitation of the study was the relatively low number of mice and few time points to be analyzed. Moreover, we only studied mice at 7 and 28 days after MI. Collagen accumulation extends with time [33], and there might be a difference in ventricular collagen content at later timepoints between WT and FAP-KO animals. Likewise, we did not detect differences in heart failure symptoms, ventricular rupture and mortality between groups. A longer study period extending 28 days post-MI might have shown a beneficial effect of genetic FAP deletion on symptoms of heart failure, ventricular rupture or mortality. In this study we only compared infarcted wild type and FAP-KO mice, but a sham group without MI is missing.

Our results do not reveal a mechanistic cause for the improved remodeling in FAP-KO mice, and future studies are needed to assess the clinical value, if any, of FAP deletion after MI. Because collagen metabolism and ventricular remodeling are different between species [34], studies of FAP depletion in larger animal models are necessary.

## Conclusion

The aim of the present study was to analyze whether FAP deficiency may have a protective effect after MI. In fact, MI in FAP-KO mice was associated with reduced LV dilation and did not negatively impact wound healing.

High left ventricular FAP signal intensities as measured by positron-emission-tomography are associated with cardiovascular and metabolic risk factors such as hypertension, diabetes mellitus and obesity [35]. Moreover, advances have been made using therapies depleting FAP cells to treat cancer disease, which could potentially affect the heart [16, 36]. Because therapies targeting FAP^pos^ (myo)fibroblasts will likely alter myocardial FAP levels, it is of importance to understand the function and pathophysiological significance of FAP deficiency in normal healthy hearts and post-MI *in vivo*.

Here, we describe for the first time that genetic ablation of FAP has beneficial effects after MI, and does not alter myocardial structure in healthy animals until 6 months of age. Our study indicates that new therapies associated with a reduction of myocardial FAP levels are safe and can be further developed. More studies are warranted to evaluate the effect of depleting FAP^pos^ cell populations on cardiac function and healing after MI in larger animal models.

## Supporting information

S1 Fig Anatomical and morphometric measurements, collagen content in healthy FAP-KO and WT mice at age of 6 months. Body weight was slightly, but significant less in FAP-KO animals compared to WT (A). However, heart weight corrected for tibia length, as well as LV area and LV cavity area (LV area/LV cavity) were not different between groups (B). Collagen content, as measured by hydroxyproline assay, showed no significant difference between groups (C). H&E stained representative examples of LV transversal sections at age 6 months (D). * = p<0.05 WT vs. FAP-KO. Plots show individual data and mean. A: WT n = 10, FAP-KO n = 19; B: WT n = 5, FAP-KO n = 9; C: WT n = 5, FAP-KO n = 5; Mann-Whitney-U test. Scale bars = 1mm.
(TIF)

S2 Fig Expression of FAP in wildtype and FAP-KO mice was analyzed by western blot. FAP was expressed in the infarct area of WT mice 7 days after myocardial infarction (MI), but not in FAP-KO mice (A). Likewise, FAP was expressed in isolated cardiac fibroblasts of WT mice, but not in FAP-KO mice (B). The monoclonal FAP-antibody detects the FAP monomer (85 kDa) and DPPIV (115 kDa) under reducing conditions, as shown previously (1). Cardiac fibroblasts isolated from hearts of healthy WT and FAP-KO animals showed no differences in cell morphology and growth (C).
(TIF)

S3 Fig Body weight, lung weight, as well as left ventricular (LV) and right ventricular (RV) weights were not different between WT (n = 8) and FAP-KO mice (n = 12) at 28 days after MI. Plots show individual data and mean. Mann-Whitney-U test.
(TIF)

S1 Raw image A) Uncropped western blot of expression of FAP in wildtype (WT) and FAP-KO (KO) mice in samples of infarct area as shown in S2A Fig. On the left panel the staining with FAP antibody is shown. The blot was flipped horizontally in S2A Fig to match lane layout (left: WT, right: KO) with S2B Fig. On the right panel staining with antibody for housekeeping gene GAPDH for loading control is shown. B) Uncropped western blot of isolated cardiac fibroblasts shown in S2B Fig. Left panel shows staining with FAP antibody. Right panel shows staining with antibody for housekeeping gene GAPDH for loading control. The western blot analysis contained also samples from wildtype HT 1080 cells (HT1080 cells wild type), FAP-overexpressing HT1080 cells (hFAP overexpressing HT 1080 cells) and recombinant human FAP- and DPPIV-Protein (recombinant human FAP/ human DPPIV) serving as controls. In S2A and S2B Fig only the groups WT and KO are shown. Antibodies used are given in S1 Material.
(PDF)

S1 Table Serial echocardiographic analysis after MI. (DOCX)

S2 Table Antibodies used for immunohistochemistry. (DOCX)

S3 Table Antibodies used for western blot. (DOCX)

S1 Material (DOCX)

## Nonstandard abbreviations

AP: Apical
ECM: Extracellular matrix
FAP: Fibroblast Activation Protein alpha
KO: Knock-Out
LV: Left ventricle
MI–: Myocardial Infarction
PA: Papillary
RV: Right ventricle
TGFβ: Transforming growth factor beta

## References

[1] Goldsmith EC, Bradshaw AD, Zile MR, Spinale FG. Myocardial fibroblast-matrix interactions and potential therapeutic targets. J Mol Cell Cardiol. 2014 5;70:92–9. 10.1016/j.yjmcc.2014.01.008 24472826 PMC4005609

[2] Cleutjens JP, Blankesteijn WM, Daemen MJ, Smits JF. The infarcted myocardium: simply dead tissue, or a lively target for therapeutic interventions. Cardiovasc Res. 1999 11;44(2):232–41. 10.1016/s0008-6363(99)00212-6 10690298

[3] van den Borne SW, Diez J, Blankesteijn WM, Verjans J, Hofstra L, Narula J. Myocardial remodeling after infarction: the role of myofibroblasts. Nat Rev Cardiol. 2010 1;7(1):30–7. 10.1038/nrcardio.2009.199 19949426

[4] Fraccarollo D, Galuppo P, Bauersachs J. Novel therapeutic approaches to post-infarction remodelling. Cardiovasc Res. 2012 5 1;94(2):293–303. 10.1093/cvr/cvs109 22387461

[5] Tillmanns J, Hoffmann D, Habbaba Y, Schmitto JD, Sedding D, Fraccarollo D, et al. Fibroblast activation protein alpha expression identifies activated fibroblasts after myocardial infarction. J Mol Cell Cardiol. 2015 10;87:194–203. 10.1016/j.yjmcc.2015.08.016 26319660

[6] Varasteh Z, Mohanta S, Robu S, Braeuer M, Li Y, Omidvari N, et al. Molecular Imaging of Fibroblast Activity After Myocardial Infarction Using a (68)Ga-Labeled Fibroblast Activation Protein Inhibitor, FAPI-04. J Nucl Med. 2019 12;60(12):1743–9. 10.2967/jnumed.119.226993 31405922 PMC6894377

[7] Brokopp CE, Schoenauer R, Richards P, Bauer S, Lohmann C, Emmert MY, et al. Fibroblast activation protein is induced by inflammation and degrades type I collagen in thin-cap fibroatheromata. Eur Heart J. 2011 11;32(21):2713–22. 10.1093/eurheartj/ehq519 21292680 PMC3205479

[8] Stein S, Weber J, Nusser-Stein S, Pahla J, Zhang HE, Mohammed SA, et al. Deletion of fibroblast activation protein provides atheroprotection. Cardiovasc Res. 2020 5 13. 10.1093/cvr/cvaa142 32402085

[9] Acharya PS, Zukas A, Chandan V, Katzenstein AL, Pure E. Fibroblast activation protein: a serine protease expressed at the remodeling interface in idiopathic pulmonary fibrosis. Hum Pathol. 2006 3;37(3):352–60. 10.1016/j.humpath.2005.11.020 16613331

[10] Levy MT, McCaughan GW, Abbott CA, Park JE, Cunningham AM, Muller E, et al. Fibroblast activation protein: a cell surface dipeptidyl peptidase and gelatinase expressed by stellate cells at the tissue remodelling interface in human cirrhosis. Hepatology. 1999 6;29(6):1768–78. 10.1002/hep.510290631 10347120

[11] Dienus K, Bayat A, Gilmore BF, Seifert O. Increased expression of fibroblast activation protein-alpha in keloid fibroblasts: implications for development of a novel treatment option. Arch Dermatol Res. 2010 9 26. 10.1007/s00403-010-1084-x 20872224

[12] Garin-Chesa P, Old LJ, Rettig WJ. Cell surface glycoprotein of reactive stromal fibroblasts as a potential antibody target in human epithelial cancers. Proc Natl Acad Sci U S A. 1990 9;87(18):7235–9. 10.1073/pnas.87.18.7235 2402505 PMC54718

[13] Cohen SJ, Alpaugh RK, Palazzo I, Meropol NJ, Rogatko A, Xu Z, et al. Fibroblast activation protein and its relationship to clinical outcome in pancreatic adenocarcinoma. Pancreas. 2008 8;37(2):154–8. 10.1097/MPA.0b013e31816618ce 18665076

[14] Rettig WJ, Garin-Chesa P, Healey JH, Su SL, Ozer HL, Schwab M, et al. Regulation and heteromeric structure of the fibroblast activation protein in normal and transformed cells of mesenchymal and neuroectodermal origin. Cancer Res. 1993 7 15;53(14):3327–35. 8391923

[15] Dolznig H, Schweifer N, Puri C, Kraut N, Rettig WJ, Kerjaschki D, et al. Characterization of cancer stroma markers: in silico analysis of an mRNA expression database for fibroblast activation protein and endosialin. Cancer Immun. 2005;5:10. 16076089

[16] Aghajanian H, Kimura T, Rurik JG, Hancock AS, Leibowitz MS, Li L, et al. Targeting cardiac fibrosis with engineered T cells. Nature. 2019 9;573(7774):430–3. 10.1038/s41586-019-1546-z 31511695 PMC6752964

[17] Riehle C, Bauersachs J. Small animal models of heart failure. Cardiovasc Res. 2019 11 1;115(13):1838–49. 10.1093/cvr/cvz161 31243437 PMC6803815

[18] Niedermeyer J, Kriz M, Hilberg F, Garin-Chesa P, Bamberger U, Lenter MC, et al. Targeted disruption of mouse fibroblast activation protein. Mol Cell Biol. 2000 2;20(3):1089–94. 10.1128/mcb.20.3.1089-1094.2000 10629066 PMC85226

[19] Fraccarollo D, Berger S, Galuppo P, Kneitz S, Hein L, Schutz G, et al. Deletion of cardiomyocyte mineralocorticoid receptor ameliorates adverse remodeling after myocardial infarction. Circulation. 2011 2 1;123(4):400–8. 10.1161/CIRCULATIONAHA.110.983023 21242479

[20] Frantz S, Hu K, Widder J, Bayer B, Witzel CC, Schmidt I, et al. Peroxisome proliferator activated-receptor agonism and left ventricular remodeling in mice with chronic myocardial infarction. Br J Pharmacol. 2004 1;141(1):9–14. 10.1038/sj.bjp.0705585 14662734 PMC1574171

[21] Vogel B, Shinagawa H, Hofmann U, Ertl G, Frantz S. Acute DNase1 treatment improves left ventricular remodeling after myocardial infarction by disruption of free chromatin. Basic Res Cardiol. 2015 3;110(2):15. 10.1007/s00395-015-0472-y 25702039

[22] Pfeffer MA, Braunwald E. Ventricular remodeling after myocardial infarction. Experimental observations and clinical implications. Circulation. 1990 4;81(4):1161–72. 10.1161/01.cir.81.4.1161 2138525

[23] Lindsey ML. Assigning matrix metalloproteinase roles in ischaemic cardiac remodelling. Nat Rev Cardiol. 2018 8;15(8):471–9. 10.1038/s41569-018-0022-z 29752454 PMC6203614

[24] Ducharme A, Frantz S, Aikawa M, Rabkin E, Lindsey M, Rohde LE, et al. Targeted deletion of matrix metalloproteinase-9 attenuates left ventricular enlargement and collagen accumulation after experimental myocardial infarction. J Clin Invest. 2000 7;106(1):55–62. 10.1172/JCI8768 10880048 PMC517910

[25] Spinale FG, Mukherjee R, Zavadzkas JA, Koval CN, Bouges S, Stroud RE, et al. Cardiac restricted overexpression of membrane type-1 matrix metalloproteinase causes adverse myocardial remodeling following myocardial infarction. J Biol Chem. 2010 9 24;285(39):30316–27. 10.1074/jbc.M110.158196 20643648 PMC2943310

[26] Dean RG, Balding LC, Candido R, Burns WC, Cao Z, Twigg SM, et al. Connective tissue growth factor and cardiac fibrosis after myocardial infarction. J Histochem Cytochem. 2005 10;53(10):1245–56. 10.1369/jhc.4A6560.2005 15956033

[27] Frantz S, Hu K, Adamek A, Wolf J, Sallam A, Maier SK, et al. Transforming growth factor beta inhibition increases mortality and left ventricular dilatation after myocardial infarction. Basic Res Cardiol. 2008 9;103(5):485–92. 10.1007/s00395-008-0739-7 18651091

[28] Kimura T, Monslow J, Klampatsa A, Leibowitz M, Sun J, Liousia M, et al. Loss of cells expressing fibroblast activation protein has variable effects in models of TGF-beta and chronic bleomycin-induced fibrosis. Am J Physiol Lung Cell Mol Physiol. 2019 8 1;317(2):L271–L82. 10.1152/ajplung.00071.2019 31188013

[29] Virag JI, Murry CE. Myofibroblast and endothelial cell proliferation during murine myocardial infarct repair. Am J Pathol. 2003 12;163(6):2433–40. 10.1016/S0002-9440(10)63598-5 14633615 PMC1892355

[30] Frantz S, Hofmann U, Fraccarollo D, Schafer A, Kranepuhl S, Hagedorn I, et al. Monocytes/macrophages prevent healing defects and left ventricular thrombus formation after myocardial infarction. FASEB J. 2013 3;27(3):871–81. 10.1096/fj.12-214049 23159933

[31] Monslow J, Todd L, Chojnowski JE, Govindaraju PK, Assoian RK, Pure E. Fibroblast Activation Protein Regulates Lesion Burden and the Fibroinflammatory Response in Apoe-Deficient Mice in a Sexually Dimorphic Manner. Am J Pathol. 2020 5;190(5):1118–36. 10.1016/j.ajpath.2020.01.004 32084369 PMC7237832

[32] Spinale FG. Myocardial matrix remodeling and the matrix metalloproteinases: influence on cardiac form and function. Physiol Rev. 2007 10;87(4):1285–342. 10.1152/physrev.00012.2007 17928585

[33] Fomovsky GM, Holmes JW. Evolution of scar structure, mechanics, and ventricular function after myocardial infarction in the rat. Am J Physiol Heart Circ Physiol. 2010 1;298(1):H221–8. 10.1152/ajpheart.00495.2009 19897714 PMC2806135

[34] Jugdutt BI, Joljart MJ, Khan MI. Rate of collagen deposition during healing and ventricular remodeling after myocardial infarction in rat and dog models. Circulation. 1996 7 1;94(1):94–101. 10.1161/01.cir.94.1.94 8964124

[35] Heckmann MB, Reinhardt F, Finke D, Katus HA, Haberkorn U, Leuschner F, et al. Relationship Between Cardiac Fibroblast Activation Protein Activity by Positron Emission Tomography and Cardiovascular Disease. Circ Cardiovasc Imaging. 2020 9;13(9):e010628. 10.1161/CIRCIMAGING.120.010628 32912030 PMC7497888

[36] Pure E, Blomberg R. Pro-tumorigenic roles of fibroblast activation protein in cancer: back to the basics. Oncogene. 2018 8;37(32):4343–57. 10.1038/s41388-018-0275-3 29720723 PMC6092565

