# Supplementary figures and images for: Genetic ablation of fibroblast activation protein alpha attenuates left ventricular dilation after myocardial infarction
Source: PLoS One. 2021 Mar 5;16(3):e0248196. doi: 10.1371/journal.pone.0248196 (PMC7935287; doi:10.1371/journal.pone.0248196)

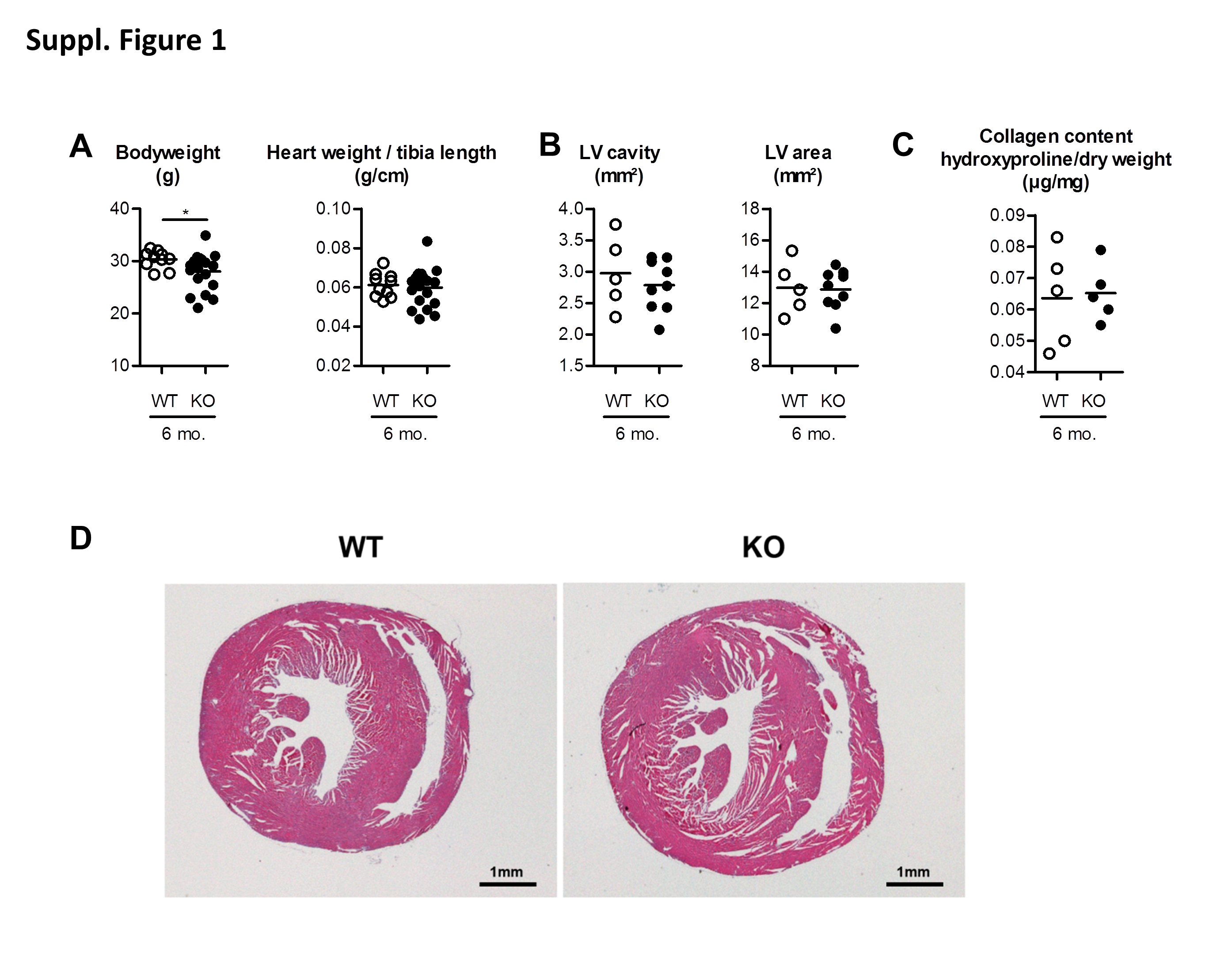

Supplement: S1 Fig — Body weight was slightly, but significant less in FAP-KO animals compared to WT (A). However, heart weight corrected for tibia length, as well as LV area and LV cavity area (LV area/LV cavity) were not different between groups (B). Collagen content, as measured by hydroxyproline assay, showed no significant difference between groups (C). H&E stained representative examples of LV transversal sections at age 6 months (D). * = p<0.05 WT vs. FAP-KO. Plots show individual data and mean. A: WT n = 10, FAP-KO n = 19; B: WT n = 5, FAP-KO n = 9; C: WT n = 5, FAP-KO n = 5; Mann-Whitney-U test. Scale bars = 1mm. (TIF) [file pone.0248196.s001.tif]

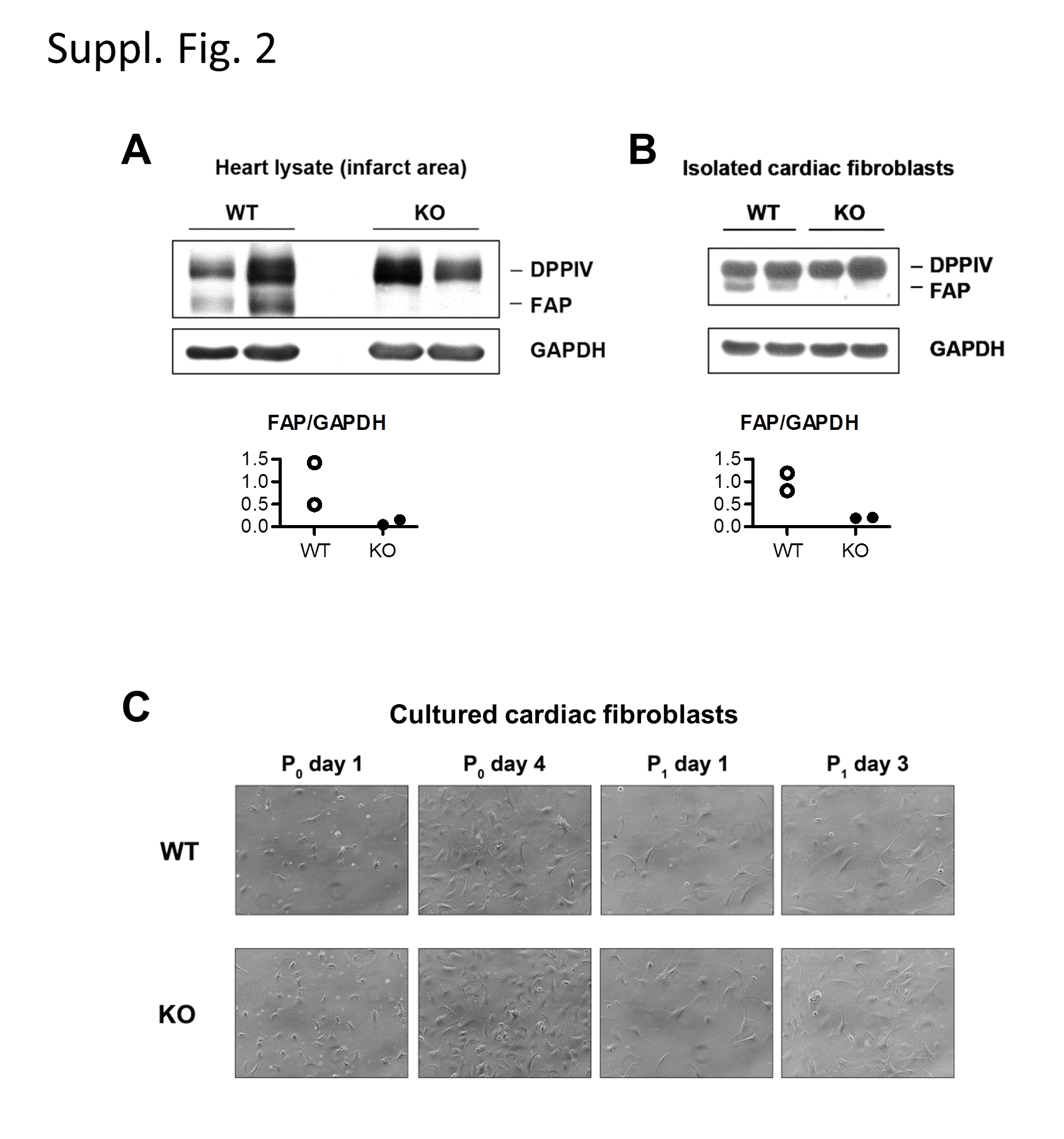

Supplement: S2 Fig — FAP was expressed in the infarct area of WT mice 7 days after myocardial infarction (MI), but not in FAP-KO mice (A). Likewise, FAP was expressed in isolated cardiac fibroblasts of WT mice, but not in FAP-KO mice (B). The monoclonal FAP-antibody detects the FAP monomer (85 kDa) and DPPIV (115 kDa) under reducing conditions, as shown previously (1). Cardiac fibroblasts isolated from hearts of healthy WT and FAP-KO animals showed no differences in cell morphology and growth (C). (TIF) [file pone.0248196.s002.tif]

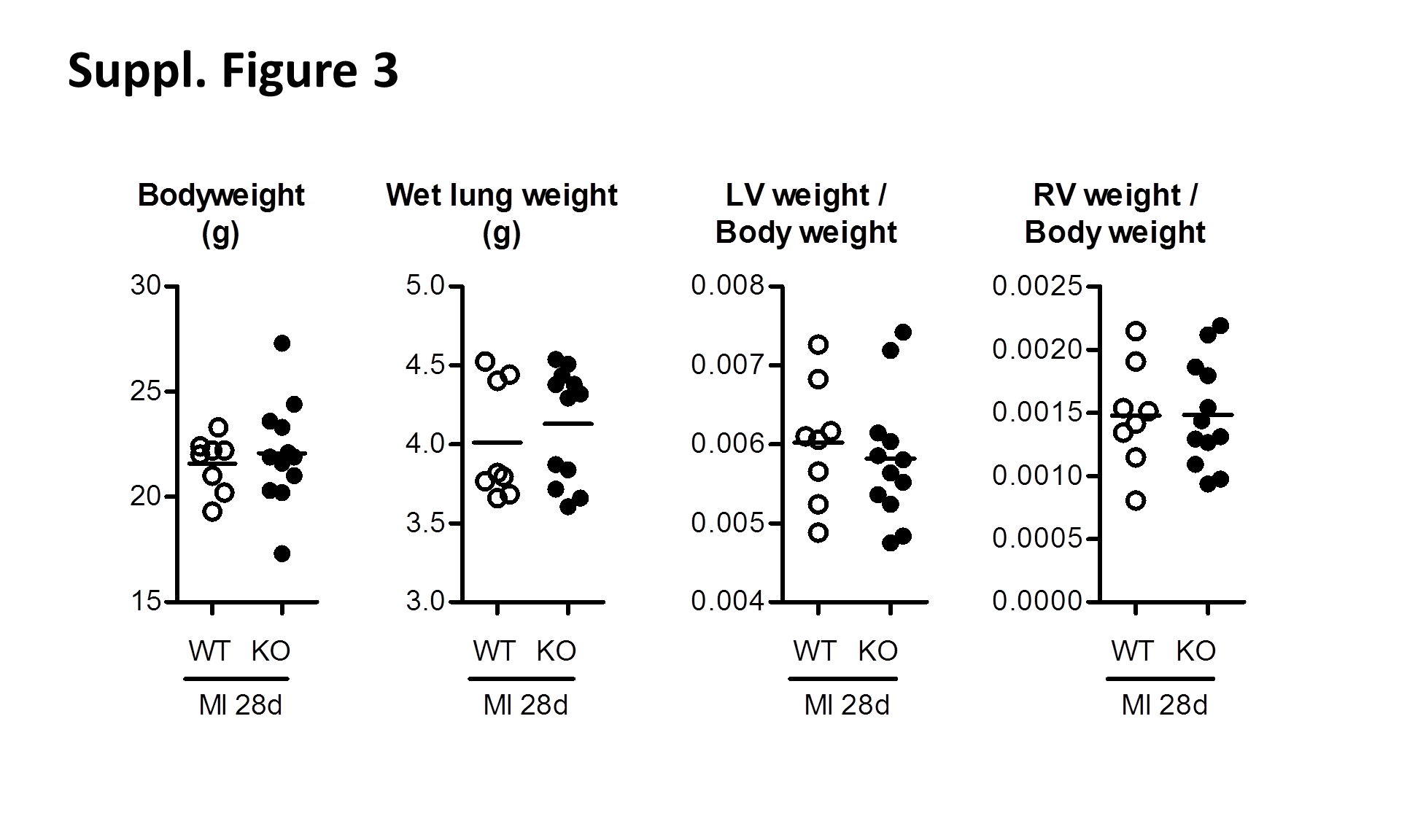

Supplement: S3 Fig — Plots show individual data and mean. Mann-Whitney-U test. (TIF) [file pone.0248196.s003.tif]

# A

## FAP

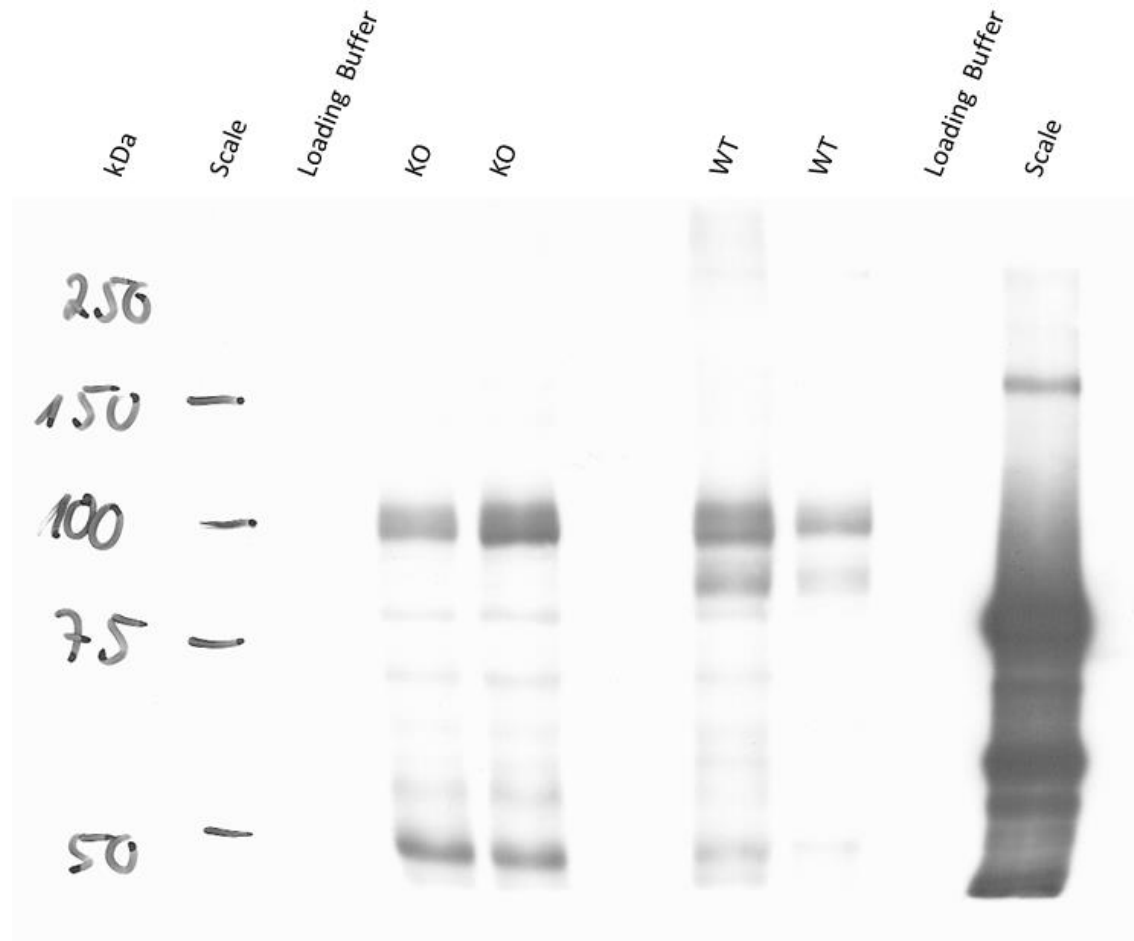

## GAPDH

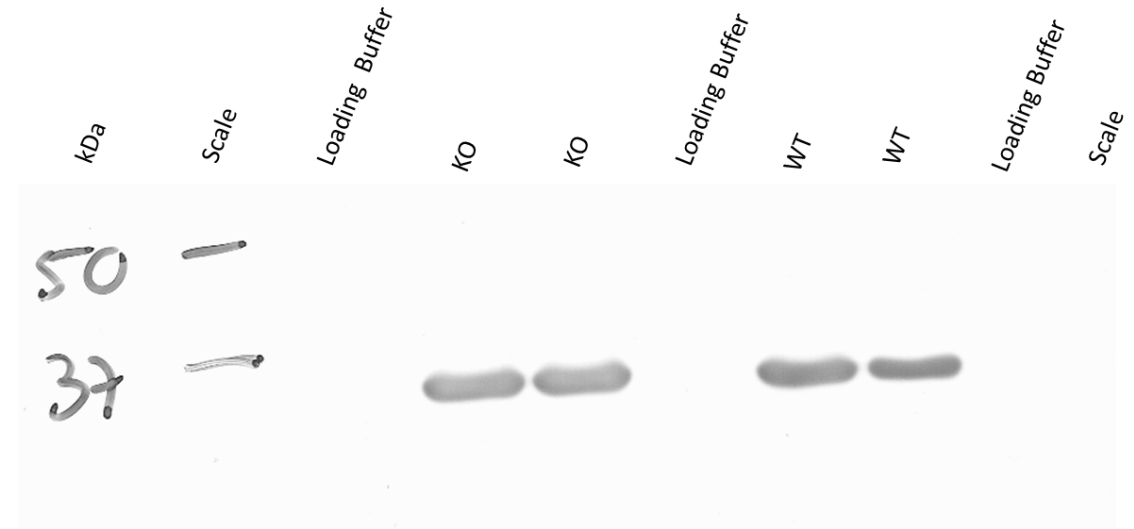

B

FAP

GAPDH

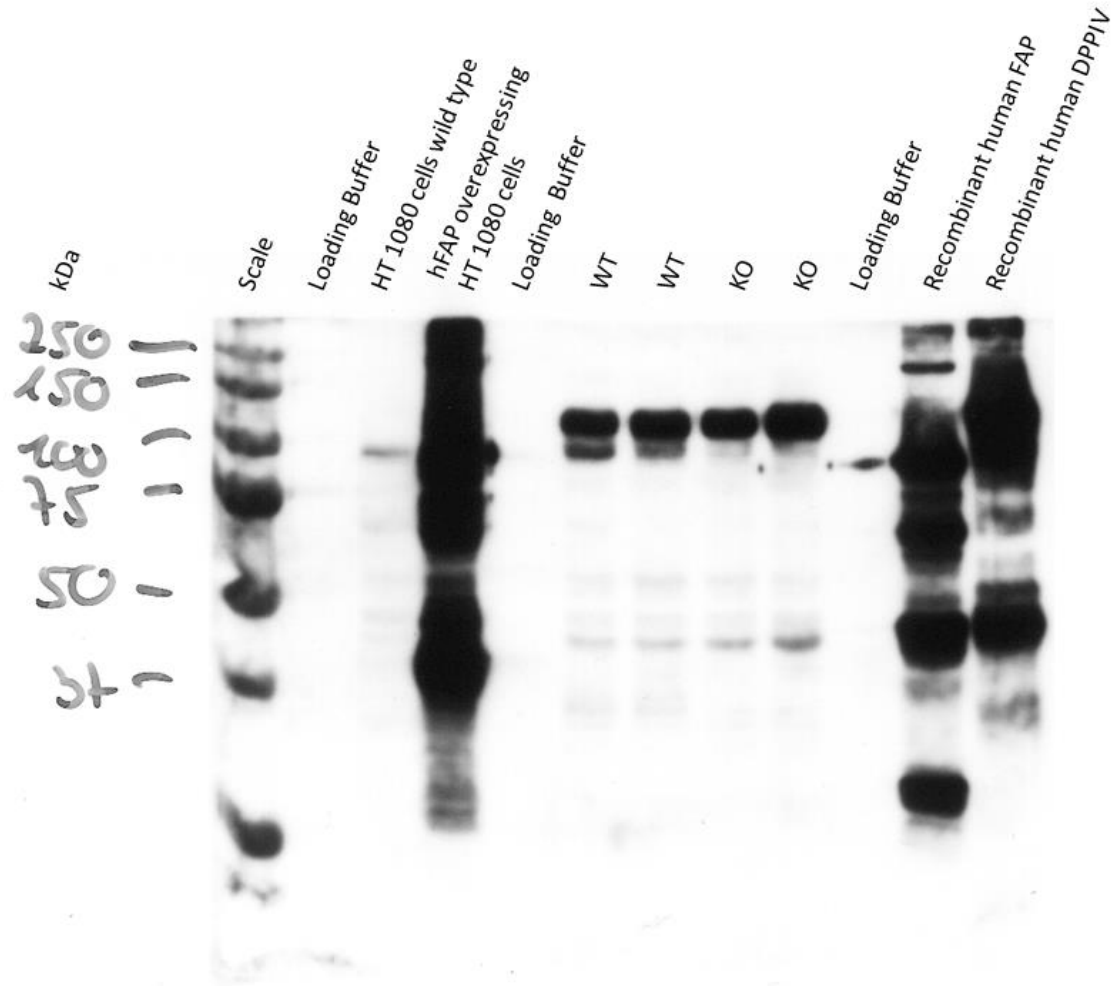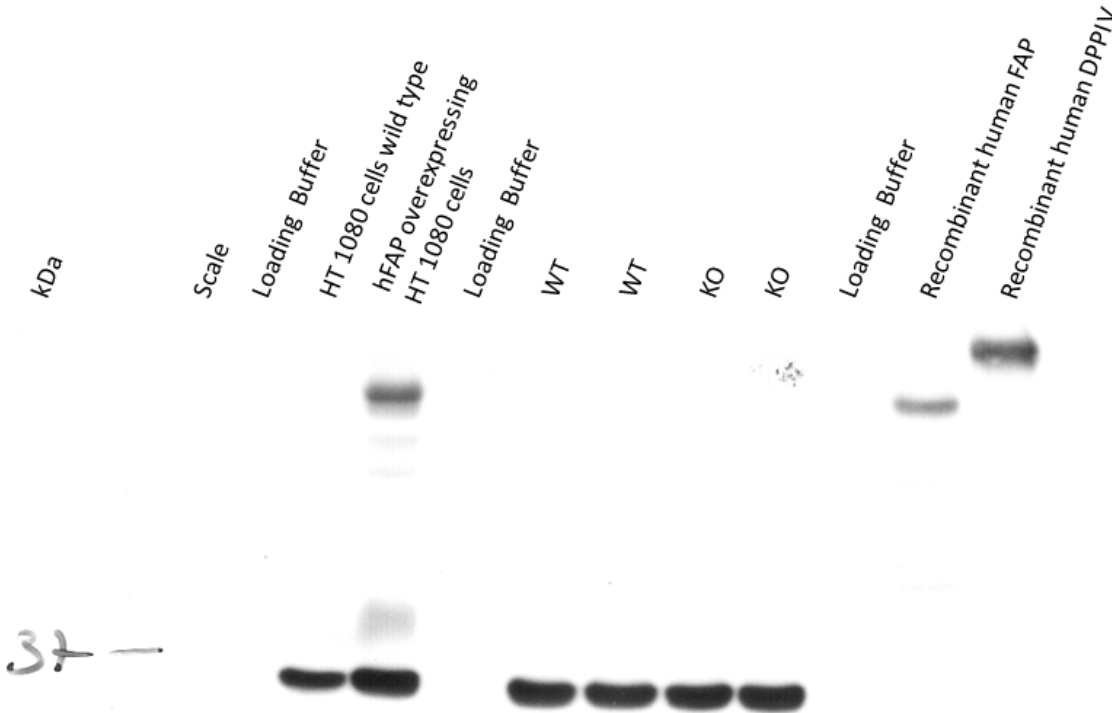

Supplement: S1 Raw image — A) Uncropped western blot of expression of FAP in wildtype (WT) and FAP-KO (KO) mice in samples of infarct area as shown in S2A Fig. On the left panel the staining with FAP antibody is shown. The blot was flipped horizontally in S2A Fig to match lane layout (left: WT, right: KO) with S2B Fig. On the right panel staining with antibody for housekeeping gene GAPDH for loading control is shown. B) Uncropped western blot of isolated cardiac fibroblasts shown in S2B Fig. Left panel shows staining with FAP antibody. Right panel shows staining with antibody for housekeeping gene GAPDH for loading control. The western blot analysis contained also samples from wildtype HT 1080 cells (HT1080 cells wild type), FAP-overexpressing HT1080 cells (hFAP overexpressing HT 1080 cells) and recombinant human FAP- and DPPIV-Protein (recombinant human FAP/ human DPPIV) serving as controls. In S2A and S2B Fig only the groups WT and KO are shown. Antibodies used are given in S1 Material. (PDF) [file pone.0248196.s004.pdf]
