# Supplementary material for: Genetic ablation of fibroblast activation protein alpha attenuates left ventricular dilation after myocardial infarction
Source: PLoS One. 2021 Mar 5;16(3):e0248196. doi: 10.1371/journal.pone.0248196 (PMC7935287; doi:10.1371/journal.pone.0248196)
Supplement: S1 Table — (DOCX) [file pone.0248196.s005.docx]

S1 Table: Serial echocardiographic analysis after MI.

| Day after MI | Wildtype | FAP-KO | *p* |
| --- | --- | --- | --- |
| AP EDA (mm²) |  |  |  |
| 0 | 5.42 ± 0.29 | 5.43 ± 0.31 | n.s. |
| 14 | 28.48 ± 2 | 21.88 ± 2.42 | n.s. |
| 28 | 31.63 ± 2.82 | 26.56 ± 1.83 | n.s. |
| AP ESA (mm²) |  |  |  |
| 0 | 2.29 ± 0.12 | 2.80 ± 0.23 | n.s. |
| 14 | 25.60 ± 2.28 | 20.49 ± 1.71 | n.s. |
| 28 | 28.67 ± 2.71 | 23.03 ± 1.78 | n.s. |
| AP 2D FS (%) |  |  |  |
| 0 | 0.57 ± 0.03 | 0.49 ± 0.02 | <0.05 |
| 14 | 0.11 ± 0.03 | 0.13 ± 0.02 | n.s. |
| 28 | 0.10 ± 0.02 | 0.14 ± 0.02 | n.s. |
| PA EDA (mm²) |  |  |  |
| 0 | 6.21 ± 0.33 | 6.25 ± 0.28 | n.s. |
| 14 | 28.67 ± 2.07 | 22.72 ± 1.49 | <0.05 |
| 28 | 30.54 ± 2.41 | 25.25 ± 1.66 | <0.05 |
| PA ESA (mm²) |  |  |  |
| 0 | 2.79 ± 0.18 | 3.14 ± 0.15 | n.s. |
| 14 | 24.38 ± 1.94 | 18.79 ± 1.44 | n.s. |
| 28 | 27.04 ± 2.4 | 21.44 ± 1.72 | <0.05 |
| PA 2D FS (%) |  |  |  |
| 0 | 0.55 ± 0.02 | 0.49 ± 0.02 | n.s. |
| 14 | 0.15 ± 0.01 | 0.18 ± 0.02 | n.s. |
| 28 | 0.12 ± 0.01 | 0.16 ± 0.02 | n.s. |
| Heart rate |  |  |  |
| 0 | 600 ± 13 | 594 ± 11 | n.s. |
| 14 | 548 ± 8 | 551 ± 19 | n.s. |
| 28 | 564 ± 17 | 546 ± 22 | n.s. |

Following echocardiographic measurements were performed at the mid-papillary (PA) muscle level and at the apical (AP) third of the ventricle: Apical/papillary end-diastolic area (AP/PA EDA), apical/papillary end-systolic area (AP/PA ESA), apical/papillary 2-dimensional fractional shortening (AP/PA 2D FS)(B-Mode). WT n=8, FAP-KO n=12, Mann-Whitney-U test WT vs. FAP-KO.
