# Supplementary material for: Genetic ablation of fibroblast activation protein alpha attenuates left ventricular dilation after myocardial infarction
Source: PLoS One. 2021 Mar 5;16(3):e0248196. doi: 10.1371/journal.pone.0248196 (PMC7935287; doi:10.1371/journal.pone.0248196)
Supplement: S2 Table — (DOCX) [file pone.0248196.s006.docx]

## **S2 Table Antibodies used for immunohistochemistry**

| **Protein** | **Antibody** | **Manufacturer** | **Labeling** | **Chromogen^1^** |
| --- | --- | --- | --- | --- |
| α-smooth muscle actin | Rabbit polyclonal | Abcam | Chromogenic | DAB |
| CD68 | Rat monoclonal | Abd Serotec | Chromogenic | DAB |
| CD31 | Rat monoclonal | Dianova | Chromogenic | DAB |

^1^ Abbreviations: DAB, 3,3'-Diaminobenzidine
