# Supplementary material for: Genetic ablation of fibroblast activation protein alpha attenuates left ventricular dilation after myocardial infarction
Source: PLoS One. 2021 Mar 5;16(3):e0248196. doi: 10.1371/journal.pone.0248196 (PMC7935287; doi:10.1371/journal.pone.0248196)
Supplement: S3 Table — (DOCX) [file pone.0248196.s007.docx]

## **S3 Table Antibodies used for Western blot**

| **Protein** | **Antibody** | **Manufacturer** | **Tissue** |
| --- | --- | --- | --- |
| GAPDH | Mouse monoclonal | Abcam | Cardiac fibroblast lysate, mouse LV |
| FAP | Mouse monoclonal | Abcam | Cardiac fibroblast lysate, mouse LV |
