# Supplementary material for: Genetic ablation of fibroblast activation protein alpha attenuates left ventricular dilation after myocardial infarction
Source: PLoS One. 2021 Mar 5;16(3):e0248196. doi: 10.1371/journal.pone.0248196 (PMC7935287; doi:10.1371/journal.pone.0248196)
Supplement: S1 Material — (DOCX) [file pone.0248196.s008.docx]

# S1 Material.

## **Morphometrical analysis of mouse myocardial tissue**

Formalin fixed, paraffin embedded LV short-axis sections of 4µm thickness from the mid portion of the LV, at papillary muscle level, were stained with Hematoxylin and eosin and imaged using a Nikon NiE microscope with motorized stage at 10x magnification to include the entire section of the heart. NIS Elements 4.2 AR software (Nikon Instruments) was used to trace the areas of the left ventricle (LV area), the infarct zone and surviving myocardium. LV cavity area was measured as area enclosed by LV endocardial circumference. Scar thickness in infarcted hearts was measured at the thinnest point and additionally as the average length of three segments from the center of the left ventricle through the scar. Septal wall thickness was measured by taking the average length of three segments from the center of the left ventricle through the septum. To quantitate both the degree of left ventricular dilation and the degree of infarct wall thinning, infarct expansion index was calculated as [(LV cavity Area / total LV Area) x (Septum thickness / avg. scar thickness)] as described previously (2-5). One representative section from the LV per animal was analyzed.

## **Immunohistochemistry of mouse myocardial tissue**

At 7 days after MI, mouse hearts were washed in saline and separated left ventricles were embedded in OCT compound (Tissue-Tek) and frozen for subsequent analyses. Short-axis sections of 4µm thickness were fixed with ice-cold acetone. A blocking-step using 10% donkey serum in PBS was followed by sequential incubation with primary antibodies against CD68 or α smooth muscle actin (SMA, S2 Table), and then by biotinylated secondary antibodies (Vector, Burlingame CA, USA), avidin-biotin-complex (Vector) and 3,3'-Diaminobenzidine (DAB, Abcam, Cambridge, UK) as chromogenic substrate.

Quantification of marker expression was performed for CD68 and SMA within infarcted and surviving myocardium of the LV at 7 days after MI using images taken at 10x magnification with a Nikon NiE microscope with motorized stage to include the entire section. For expression analysis, a manual color threshold was applied to separate stained and background areas, and stained area fraction was determined as stained vs. total tissue area for each image using NIS Elements 4.2 AR software (Nikon Instruments) (6,7). Image processing with Photoshop (Adobe) included changes in brightness, contrast and tonal range, and was applied equally across the entire image.

## **Capillary density**

At 28 days after MI, hearts were washed in saline and separated left ventricles were preserved in formalin. Formalin fixed, paraffin embedded tissues where sectioned at 4 µm, and heat induced antigen retrieval was performed using Histosafe Enhancer (Linaris, Germany). After blocking with 10% horse serum, sections were incubated with anti-CD31 primary antibody (S2 Table), followed by biotinylated secondary antibodies (Vector, Burlingame CA, USA), avidin-biotin-complex (Vector) and 3,3'-Diaminobenzidine (DAB, Abcam, Cambridge, UK) as chromogenic substrate. Images were obtained at 20x with a Nikon NiE microscope, and capillaries within the surviving myocardium were quantified using Image Pro Plus software (Media Cybernetics, Bethesda, USA).

## **In vitro studies with cardiac fibroblasts**

12 week old healthy male mice were sacrificed, the hearts immediately removed, washed in saline, gently minced into small tissue pieces, followed by digestion using collagenase type II (1mg/ml, Worthington) in Leibovitz L-15 medium supplemented with L-Glutamine (Lonza) for 2 hours at 37°C. After digestion and repeated gentle aspiration using a transfer pipette, cells were filtered through 100µm and 40µm cell strainers and cultured for 1 passage in DMEM supplemented with 10% frozen bovine serum, 4.5 g/l glucose, 10mM HEPES and antibiotics (Lonza).

## **Western Blot of mouse myocardial tissue and cardiac fibroblasts**

To show increased FAP expression in wild type mice, and diminished FAP expression in FAP deficient animals we performed western blot analysis because it allows validation by molecular weight of the protein band. For Western blot analysis of mouse hearts 7 days after MI, the LV was divided into infarcted and noninfarcted myocardium including septum. LV samples were homogenized in ice-cold radioimmunoprecipitation assay (RIPA) buffer as described previously (7). For cultured fibroblasts, cells were lysed using ice-cold cell lysis buffer (Cell Signaling). Proteins were electrotransferred overnight onto polyvinylidene difluoride membrane (Immun-Blot, Bio-Rad) and stained using antibodies against FAP (S3 Table). Western blot was performed under reducing conditions, and FAP was detected at ~90kDa corresponding to the FAP monomer (7). The bands were detected using chemiluminescence assay (ECL or ECL plus, GE Healthcare Life Sciences). Image processing with Photoshop (Adobe) included horizontal flip, changes in brightness, contrast and tonal range, and was applied equally across the entire gel image.

## **Analysis of collagen content after MI**

To assess scar collagen content, LV sections were stained with picrosirius red and examined using a Nikon NiE microscope at 20x magnification. Tissue images were analyzed using SigmaScanPro 5.0 image analysis software (Systat Software Inc). Collagen stained area was expressed as a percentage of the area of each image (8).

## **Hydroxyproline determination in aged healthy mice**

Hearts of 6 months old WT and FAP-KO male mice were removed, washed in saline and homogenized (1:30 w / v) in ice-cold extraction buffer (pH 5.0) containing cacodylic acid (10mmol/l), NaCl (0.15 mmol/l), ZnCl (1mmol/l), CaCl (20mmol/l), NaN3 (1.5 mmol/l), and 0.01% Triton X-100. Tissue homogenates were freeze–dried, weighed and hydrolyzed in 6N HCl at 110 °C for 24h. Hydroxyproline concentration was measured spectrophotometrically (4). Collagen content was expressed in µg/mg dry tissue weight assuming that collagen contains an average 13.4% in hydroxyproline. Assays were performed in triplicate.

## **References in supporting information**

1. Tillmanns J, Widera C, Habbaba Y, Galuppo P, Kempf T, Wollert KC, et al. Circulating concentrations of fibroblast activation protein alpha in apparently healthy individuals and patients with acute coronary syndrome as assessed by sandwich ELISA. Int J Cardiol. 2013;168(4):3926-31

2. Hochman JS, Choo H. Limitation of myocardial infarct expansion by reperfusion independent of myocardial salvage. Circulation. 1987;75(1):299-306.

3. Virag JI, Murry CE. Myofibroblast and endothelial cell proliferation during murine myocardial infarct repair. Am J Pathol. 2003;163(6):2433-40.

4. Fraccarollo D, Bauersachs J, Kellner M, Galuppo P, Ertl G. Cardioprotection by long-term ET(A) receptor blockade and ACE inhibition in rats with congestive heart failure: mono- versus combination therapy. Cardiovasc Res. 2002;54(1):85-94.

5. Fraccarollo D, Berger S, Galuppo P, Kneitz S, Hein L, Schutz G, et al. Deletion of cardiomyocyte mineralocorticoid receptor ameliorates adverse remodeling after myocardial infarction. Circulation. 2011;123(4):400-8.

6. Leal S, Diniz C, Sa C, Goncalves J, Soares AS, Rocha-Pereira C, et al. Semiautomated computer-assisted image analysis to quantify 3,3'-diaminobenzidine tetrahydrochloride-immunostained small tissues. Anal Biochem. 2006;357(1):137-43.

7. Tillmanns J, Hoffmann D, Habbaba Y, Schmitto JD, Sedding D, Fraccarollo D, et al. Fibroblast activation protein alpha expression identifies activated fibroblasts after myocardial infarction. J Mol Cell Cardiol. 2015;87:194-203.

8. Thum T, Gross C, Fiedler J, Fischer T, Kissler S, Bussen M, et al. MicroRNA-21 contributes to myocardial disease by stimulating MAP kinase signalling in fibroblasts. Nature. 2008;456(7224):980-4.
